# Supplementary material for: Single-cell CAR T atlas reveals type 2 function in 8-year leukaemia remission
Source: Nature. 2024 Sep 25;634(8034):702–11. doi: 10.1038/s41586-024-07762-w (PMC11485231; doi:10.1038/s41586-024-07762-w)
Supplement: Supplementary file 1 — Supplementary Fig. 1: the representative flow cytometry gating strategy. [file 41586_2024_7762_MOESM1_ESM.pdf]

nature portfolio

https://doi.org/10.1038/s41586-024-07762-w

Supplementary information

Single-cell CAR T atlas reveals type 2 function in 8-year leukaemia remission

In the format provided by the authors and unedited

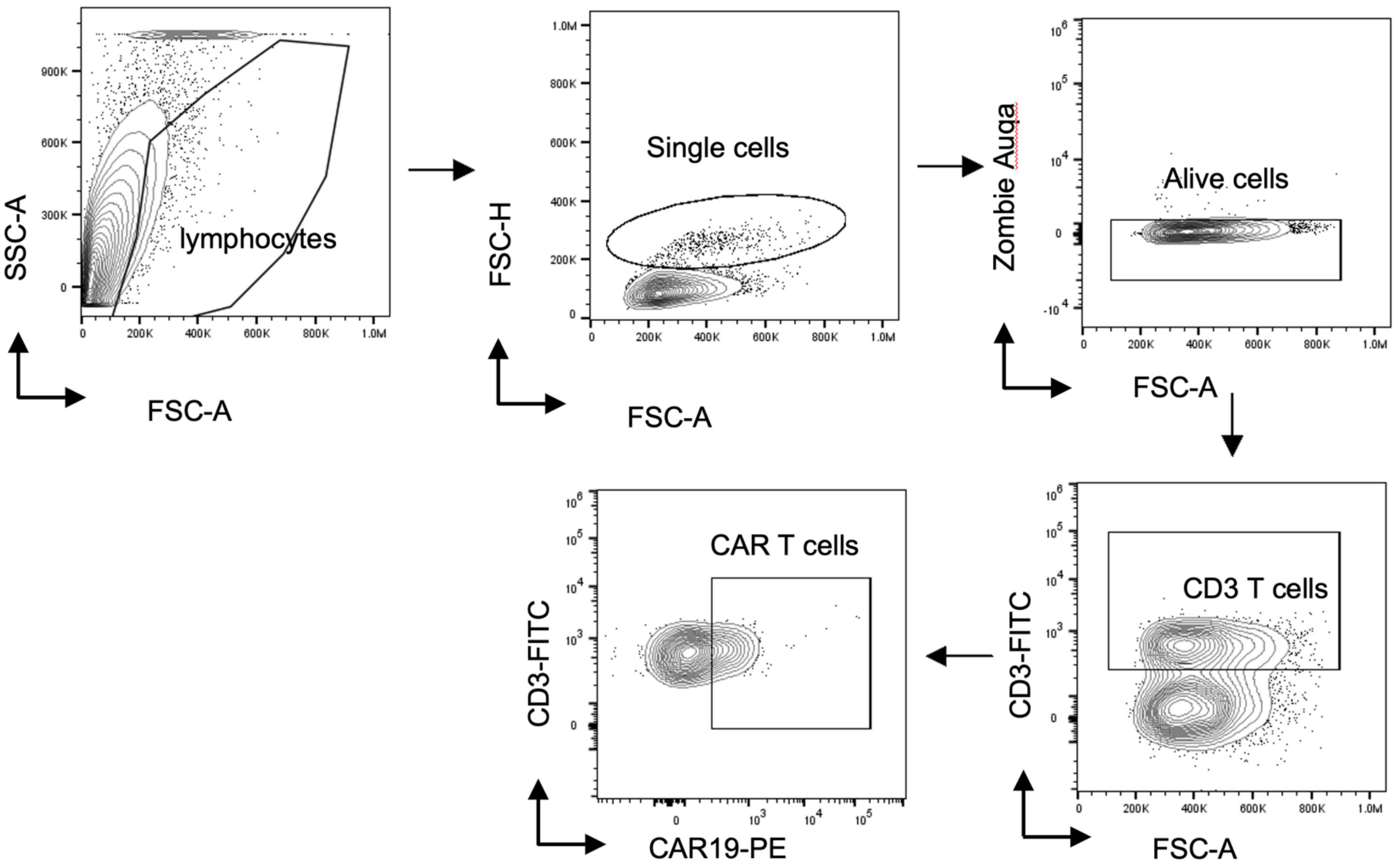

Supplementary Fig. 1 | Representative flow cytometry gating strategy.

Gating strategy employed for flow cytometry data analysis corresponding to Extended Data Fig. 12 (g, h), Extended Data Fig. 13 (c–g, j), and Extended Data Fig. 14 (e–g, i, j).
