# Supplementary material for: Single-cell CAR T atlas reveals type 2 function in 8-year leukaemia remission
Source: Nature. 2024 Sep 25;634(8034):702–11. doi: 10.1038/s41586-024-07762-w (PMC11485231; doi:10.1038/s41586-024-07762-w)
Supplement: Supplementary file 2 — Reporting Summary [file 41586_2024_7762_MOESM2_ESM.pdf]

nature portfolio

Corresponding author(s): Rong Fan, Stephan A. Grupp, J. Joseph Melenhorst, Carl H. June, and Li Tang

Last updated by author(s): Jun 10, 2024

Reporting Summary

Nature Portfolio wishes to improve the reproducibility of the work that we publish. This form provides structure for consistency and transparency in reporting. For further information on Nature Portfolio policies, see our Editorial Policies and the Editorial Policy Checklist.

Statistics

For all statistical analyses, confirm that the following items are present in the figure legend, table legend, main text, or Methods section.

n/a	Confirmed	
☐	☒	The exact sample size (*n*) for each experimental group/condition, given as a discrete number and unit of measurement
☐	☒	A statement on whether measurements were taken from distinct samples or whether the same sample was measured repeatedly
☐	☒	The statistical test(s) used AND whether they are one- or two-sided<br>*Only common tests should be described solely by name; describe more complex techniques in the Methods section.*
☒	☐	A description of all covariates tested
☐	☒	A description of any assumptions or corrections, such as tests of normality and adjustment for multiple comparisons
☐	☒	A full description of the statistical parameters including central tendency (e.g. means) or other basic estimates (e.g. regression coefficient) AND variation (e.g. standard deviation) or associated estimates of uncertainty (e.g. confidence intervals)
☐	☒	For null hypothesis testing, the test statistic (e.g. *F*, *t*, *r*) with confidence intervals, effect sizes, degrees of freedom and *P* value noted<br>*Give P values as exact values whenever suitable.*
☒	☐	For Bayesian analysis, information on the choice of priors and Markov chain Monte Carlo settings
☒	☐	For hierarchical and complex designs, identification of the appropriate level for tests and full reporting of outcomes
☐	☒	Estimates of effect sizes (e.g. Cohen's *d*, Pearson's *r*), indicating how they were calculated

Our web collection on statistics for biologists contains articles on many of the points above.

Software and code

Policy information about availability of computer code

Data collection	1. The scRNA-seq libraries were prepared using the Chromium Single-Cell 3' Library and Gel Bead Kit v3.1 (10x Genomics, Cat# PN-1000268).<br>2. Single-cell ATAC+Gene co-profiling was performed using the Chromium Next GEM Single Cell Multiome ATAC + Gene Expression kit (10x Genomics, Cat# PN-1000283).<br>3. For flow cytometry analysis of CD19-3T3 stimulated CAR T cells from 82 ALL patients, data were collected using a Cytek Aurora flow cytometer.<br>4. The multiplexed secretomic assay was performed using a IsoLight (IsoPlexis) machine.<br>5. For flow cytometry analysis of CAR T cells isolated from mouse peripheral blood, data were collected using an Attune NxT Flow Cytometer with Attune NxT Software v.3 (Invitrogen).
Data analysis	1. Single-cell transcriptome data processing and analysis: The sequencing data underwent alignment to the GRCh38 human reference genome, followed by barcode and unique molecular identifier counting, ultimately generating a digital gene expression matrix using Cell Ranger v6.1.2 (10x Genomics). The subsequent data analysis was conducted according to the Seurat v4 pipeline. The hashtag oligos expression was used to demultiplex cells back to their original sample-of-origin, while also identifying and excluding cross-sample doublets. Cells flagged as doublets (two barcodes detected) or lacking barcodes were omitted from the analysis. Only cells expressing a gene count ranging from 200 to 7,000 and exhibiting less than 10% mitochondrial gene content were retained for downstream analysis.<br>2. Single-cell ATAC+Gene co-profiling data processing and analysis: The Cell Ranger ARC v2.0.2 (10x Genomics) was utilized to perform sample demultiplexing, barcode processing, identification of open chromatin regions, and simultaneous counting of transcripts and peak accessibility in single cells from the sequenced data. The output per barcode matrices underwent joint RNA and ATAC analysis using Signac v1.12.0 and Seurat v4. Quality filtering criteria adhered to default settings. Specifically, cells were retained if they exhibited an ATAC peak count ranging from 1,000 to 100,000, a gene count ranging from 1,000 to 25,000, a nucleosome_signal below 2, and a TSS enrichment score exceeding 1. To enhance the accuracy of peak identification, we employed MACS2 v2.2.9.1 with the "CallPeaks" function.

April 2023

3. Flow cytometry analysis: Data acquired from the Cytek Aurora flow cytometer was analzyed using FlowJo v10.8.0. Data acquired from the Attune NxT Flow Cytometer was analzyed using FlowJo v10.6.1 (Tree Star).
4. The fluorescent signals in multiplexed secretomic assay were analyzed by the IsoSpeak v2.8.1.0 (IsoPlexis) software.
5. Ligand-receptor interaction analysis was performed using the R toolkit Connectome v1.0.0.
6. Ingenuity Pathway Analysis (IPA, QIAGEN) was used to reveal the underlying signaling pathways.
7. Statistical analyses were performed with Prism v10 (GraphPad) or R v4.3.1.

For manuscripts utilizing custom algorithms or software that are central to the research but not yet described in published literature, software must be made available to editors and reviewers. We strongly encourage code deposition in a community repository (e.g. GitHub). See the Nature Portfolio guidelines for submitting code & software for further information.

Data

Policy information about availability of data

All manuscripts must include a data availability statement. This statement should provide the following information, where applicable:

- Accession codes, unique identifiers, or web links for publicly available datasets
- A description of any restrictions on data availability
- For clinical datasets or third party data, please ensure that the statement adheres to our policy

Raw and processed single-cell sequencing data for this study can be accessed in the NCBI Gene Expression Omnibus (GEO) database under the accession number GSE262072.

Research involving human participants, their data, or biological material

Policy information about studies with human participants or human data. See also policy information about sex, gender (identity/presentation), and sexual orientation and race, ethnicity and racism.

Reporting on sex and gender	Sex and/or gender was not considered in this study.
Reporting on race, ethnicity, or other socially relevant groupings	Race, ethnicity, or other socially relevant groupings were not considered in this study.
Population characteristics	The detailed information was provided in the supplementary table 1. We did not perform covariate analysis in this secondary correlation investigation.
Recruitment	Pre-infusion CAR T samples were acquired from patients with relapsed/refractory B-ALL who enrolled in a Phase I/IIA pilot clinical trial designed to assess the safety and feasibility of CTL019 T cell therapy (ClinicalTrials.gov number, NCT01626495), or a pilot study of the tocilizumab optimization timing for CART19 associated cytokine release syndrome (ClinicalTrials.gov number, NCT02906371).
Ethics oversight	The current study is a secondary investigation using patient samples collected from an existing clinical trial for which the University of Pennsylvania Institutional Board provided insight.

Note that full information on the approval of the study protocol must also be provided in the manuscript.

Field-specific reporting

Please select the one below that is the best fit for your research. If you are not sure, read the appropriate sections before making your selection.

☒ Life sciences ☐ Behavioural & social sciences ☐ Ecological, evolutionary & environmental sciences

For a reference copy of the document with all sections, see nature.com/documents/nr-reporting-summary-flat.pdf

Life sciences study design

All studies must disclose on these points even when the disclosure is negative.

Sample size	This study reports single-cell multiomics profiling of pre-infusion CAR T cells from 82 pediatric ALL patients and 6 healthy donors. No statistical methods were used to pre-determine sample size. Patient grouping was based on their clinical responses, with at least five patients included in each persistence group. To ensure findings are reproducible, functional in vitro study was performed with at least 3 technical replicates and leukemia mouse model study was performed with 5 animals per each group. This sample size was determined based on experience and well-established, previously published studies.
Data exclusions	There was no specific data exclusion criteria.
Replication	The characteristics of CAR T cells in this study were analyzed using various assays and independently conducted experiments at different research centers. These included single-cell RNA and CITE-seq multiomics at Yale University, single-cell ATAC+Gene co-profiling at Yale University, flow cytometry at the University of Pennsylvania, multiplexed secretomic assays at Yale University, and serum proteomic assays at the Children's Hospital of Philadelphia. Additionally, leukemia mouse model studies were performed at École Polytechnique Fédérale de

Lausanne (EPFL). For each sample pool, two independent single-cell RNA and CITE-seq libraries were prepared and sequenced. CAR T cells derived from at least 3 different patients or healthy donors were used in each experiment to ensure robust conclusions.

Randomization	Mice were randomized prior to CAR T treatment to ensure equivalent tumor burden among groups. To uncover the hallmarks of CAR T longevity, we correlated the single-cell multi-omics profiles with the duration of B-cell aplasia (BCA), a widely used pharmacodynamic measurement indicative of CAR T persistence. Consequently, we classified all patients into five persistence groups based on their clinically observed BCA duration, with no randomization performed.
Blinding	Blinding was not performed in this study design that involved deep characterization of pre-infusion CAR T cells from 82 ALL patients. All analyses were based on comparisons between two patient groups classified according to their clinically observed responses, making blinding not possible.

Reporting for specific materials, systems and methods

We require information from authors about some types of materials, experimental systems and methods used in many studies. Here, indicate whether each material, system or method listed is relevant to your study. If you are not sure if a list item applies to your research, read the appropriate section before selecting a response.

Materials & experimental systems

n/a	Involved in the study	
☐	☒	Antibodies
☐	☒	Eukaryotic cell lines
☒	☐	Palaeontology and archaeology
☐	☒	Animals and other organisms
☐	☒	Clinical data
☒	☐	Dual use research of concern
☒	☐	Plants

Methods

n/a	Involved in the study	
☒	☐	ChIP-seq
☐	☒	Flow cytometry
☒	☐	MRI-based neuroimaging

Antibodies

Antibodies used

All TotalSeq™-B anti-human antibodies were purchased from Biolegend: CD4 (RPA-T4, 300565), CD8 (SK1, 344757), CD45RA (HI100, 304161), CD45RO (UCHL1, 304257), CD62L (DREG-56, 304849), CD95 (DX2, 305653), CD127 (A019D5, 351354), CD28 (CD28.2, 302961), CD27 (O323, 302851), CCR7 (G043H7, 353249), HLA-DR (L243, 307661), CD69 (FN50, 310949), PD-1 (EH12.2H7, 329961), TIM-3 (F38-2E2, 345053), LAG-3 (11C3C65, 369337), CTLA-4 (BNI3, 369629), TIGIT (A15153G, 372727).

The following antibodies with indicated clones were used for flow cytometry analysis of CD19-3T3 stimulated CAR T cells from 82 ALL patients: PE-labeled monoclonal anti-FMC63 scFv (CAR19) (Y45, ACRO Biosystems, FM3-HPY53), CD3 (SK7, BD Biosciences, 564001), CD4 (OKT4, Biolegend, 317442), CD8a (RPA-T8, Biolegend, 301042), CD19 (HIB19, BD Biosciences, 561121), CD14 (M5E2, BD Biosciences, 561391), IL-3 (BVD3-1F9, Biolegend, 500606), IL-4 (MP4-25D2, Biolegend, 500834), IL-5 (TRFK5, Biolegend, 504306), IL-13 (JES10-5A2, Biolegend, 501916), IL-31 (1D10B31, Biolegend, 659608).

The following antibodies with indicated clones were purchased from Biolegend and used for flow cytometry analysis of CAR T cells isolated from mouse peripheral blood: CD95 (Fas) (DX2, 305624), IL-13 (JES10-5A2, 501916), CD27 (LG.3A10, 124249), CD45RO (UCHL1, 304238), TNF-α (MAb11, 502940), CD3 (OKT3, 317306), Granzyme B (GB11, 515403), CD223 (LAG-3) (11C3C65, 369312), CD366 (TIM-3) (F38-2E2, 345016), CD197 (CCR7) (G043H7, 353235), IL-4 (MP4-25D2, 500832), CD19 (HIB19, 302216), CD4 (OKT4, 317416), IL-5 (TRFK5, 504306), CD8 (SK1, 344724), CD279 (PD-1) (EH12.2H7, 329952), KLRG1 (MAFA) (2F1/KLRG1, 138426), IFN-γ (4S.B3, 502530), and Zombie Aqua™ Fixable Viability Kit (423102). Monoclonal Anti-FMC63 Antibody (Y45, FM3-HPY53) was purchased from ACRO Biosystems.

Validation

For TotalSeq™-B antibodies, per manufacturer's website (https://www.biolegend.com/en-us/quality/quality-control): "Bulk lots are tested by PCR and sequencing to confirm the oligonucleotide barcodes. They are also tested by flow cytometry to ensure the antibodies recognize the proper cell populations. Bottled lots are tested by PCR and sequencing to confirm the oligonucleotide barcodes". Detailed validation information for each antibody is available at the following sites:

1. TotalSeq™-B0072 anti-human CD4 Antibody: https://www.biolegend.com/en-us/products/totalseq-b0072-anti-human-cd4-antibody-16820
2. TotalSeq™-B0046 anti-human CD8 Antibody: https://www.biolegend.com/en-us/products/totalseq-b0046-anti-human-cd8-antibody-18042
3. TotalSeq™-B0063 anti-human CD45RA Antibody: https://www.biolegend.com/en-us/products/totalseq-b0063-anti-human-cd45ra-antibody-16850
4. TotalSeq™-B0087 anti-human CD45RO Antibody: https://www.biolegend.com/en-us/products/totalseq-b0087-anti-human-cd45ro-antibody-16853
5. TotalSeq™-B0147 anti-human CD62L Antibody: https://www.biolegend.com/en-us/products/totalseq-b0147-anti-human-cd62l-antibody-16892
6. TotalSeq™-B0156 anti-human CD95 (Fas) Antibody: https://www.biolegend.com/en-us/products/totalseq-b0156-anti-human-cd95-fas-antibody-18636
7. TotalSeq™-B0390 anti-human CD127 (IL-7Rα) Antibody: https://www.biolegend.com/en-us/products/totalseq-b0390-anti-human-cd127-il-7ralpha-antibody-16859
8. TotalSeq™-B0386 anti-human CD28 Antibody: https://www.biolegend.com/en-us/products/totalseq-b0386-anti-human-cd28-

antibody-16842
9. TotalSeq™-B0154 anti-human CD27 Antibody: https://www.biolegend.com/en-us/products/totalseq-b0154-anti-human-cd27-antibody-16839
10. TotalSeq™-B0148 anti-human CD197 (CCR7) Antibody: https://www.biolegend.com/en-us/products/totalseq-b0148-anti-human-cd197-ccr7-antibody-16857
11. TotalSeq™-B0159 anti-human HLA-DR Antibody: https://www.biolegend.com/en-us/products/totalseq-b0159-anti-human-hla-dr-antibody-16879
12. TotalSeq™-B0146 anti-human CD69 Antibody: https://www.biolegend.com/en-us/products/totalseq-b0146-anti-human-cd69-antibody-16873
13. TotalSeq™-B0088 anti-human CD279 (PD-1) Antibody: https://www.biolegend.com/en-us/products/totalseq-b0088-anti-human-cd279-pd-1-antibody-16863
14. TotalSeq™-B0169 anti-human CD366 (Tim-3) Antibody: https://www.biolegend.com/en-us/products/totalseq-b0169-anti-human-cd366-tim-3-antibody-19028
15. TotalSeq™-B0152 anti-human CD223 (LAG-3) Antibody: https://www.biolegend.com/en-us/products/totalseq-b0152-anti-human-cd223-lag-3-antibody-19187
16. TotalSeq™-B0151 anti-human CD152 (CTLA-4) Antibody: https://www.biolegend.com/en-us/products/totalseq-b0151-anti-human-cd152-ctla-4-antibody-18639
17. TotalSeq™-B0089 anti-human TIGIT (VSTM3) Antibody: https://www.biolegend.com/en-us/products/totalseq-b0089-anti-human-tigit-vstm3-antibody-16855

For antibodies used in flow cytometry, each antibody has been validated by the manufacturer for use to detect human species targets. Detailed validation information for each antibody is available at the following sites:

1. PE-labeled monoclonal anti-FMC63 scFv (CAR19) (Y45, ACRO Biosystems, FM3-HPY53): https://www.acrobiosystems.com/P3508-PE-Labeled-Monoclonal-Anti-FMC63-Antibody-Mouse-IgG1-%28Y45%29-%28Site-specific-conjugation%29-%28Preservative-free%29.html
2. CD3 (SK7, BD Biosciences, 564001): https://www.bdbiosciences.com/en-us/products/reagents/flow-cytometry-reagents/research-reagents/single-color-antibodies-ruo/buv395-mouse-anti-human-cd3.564001
3. CD4 (OKT4, Biolegend, 317442): https://www.biolegend.com/en-us/products/brilliant-violet-785-anti-human-cd4-antibody-7978
4. CD8a (RPA-T8, Biolegend, 301042): https://www.biolegend.com/en-us/products/brilliant-violet-650-anti-human-cd8a-antibody-7652
5. CD19 (HIB19, BD Biosciences, 561121): https://www.bdbiosciences.com/en-us/products/reagents/flow-cytometry-reagents/research-reagents/single-color-antibodies-ruo/v500-mouse-anti-human-cd19.561121
6. CD14 (M5E2, BD Biosciences, 561391): https://www.bdbiosciences.com/en-us/products/reagents/flow-cytometry-reagents/research-reagents/single-color-antibodies-ruo/v500-mouse-anti-human-cd14.561391
7. IL-3 (BVD3-1F9, Biolegend, 500606): https://www.biolegend.com/en-us/products/pe-anti-human-il-3-antibody-921
8. IL-4 (MP4-25D2, Biolegend, 500834): https://www.biolegend.com/en-us/products/apc-cyanine7-anti-human-il-4-antibody-13184
9. IL-5 (TRFK5, Biolegend, 504306): https://www.biolegend.com/en-us/products/apc-anti-mouse-human-il-5-antibody-989
10. IL-13 (JES10-5A2, Biolegend, 501916): https://www.biolegend.com/en-us/products/brilliant-violet-421-anti-human-il-13-antibody-13228
11. IL-31 (1D10B31, Biolegend, 659608): https://www.biolegend.com/en-us/products/alexa-fluor-488-anti-human-il-31-antibody-13170
12. CD95 (Fas) (DX2, 305624): https://www.biolegend.com/en-us/products/brilliant-violet-421-anti-human-cd95-fas-antibody-7252
13. IL-13 (JES10-5A2, 501916): https://www.biolegend.com/en-us/products/brilliant-violet-421-anti-human-il-13-antibody-13228
14. CD27 (LG.3A10, 124249): https://www.biolegend.com/en-us/products/brilliant-violet-605-anti-mouserathuman-cd27-antibody-19163
15.CD45RO (UCHL1, 304238): https://www.biolegend.com/en-us/products/brilliant-violet-605-anti-human-cd45ro-antibody-8569
16. TNF-α (MAb11, 502940): https://www.biolegend.com/en-us/products/brilliant-violet-711-anti-human-tnf-alpha-antibody-9034
17. CD3 (OKT3, 317306): https://www.biolegend.com/en-us/products/fitc-anti-human-cd3-antibody-3644
18. Granzyme B (GB11, 515403): https://www.biolegend.com/en-us/products/fitc-anti-human-mouse-granzyme-b-antibody-6066
19. CD223 (LAG-3) (11C3C65, 369312): https://www.biolegend.com/en-us/products/percp-cyanine5-5-anti-human-cd223-lag-3-antibody-13552
20. CD366 (TIM-3) (F38-2E2, 345016): https://www.biolegend.com/en-us/products/percp-cyanine5-5-anti-human-cd366-tim-3-antibody-8438
21. CD197 (CCR7) (G043H7, 353235): https://www.biolegend.com/en-us/products/pe-dazzle-594-anti-human-cd197-ccr7-antibody-9811
22. IL-4 (MP4-25D2, 500832): https://www.biolegend.com/en-us/products/pe-dazzle-594-anti-human-il-4-antibody-10216
23. CD19 (HIB19, 302216): https://www.biolegend.com/en-us/products/pe-cyanine7-anti-human-cd19-antibody-1911
24. CD4 (OKT4, 317416): https://www.biolegend.com/en-us/products/apc-anti-human-cd4-antibody-3657
25. IL-5 (TRFK5, 504306): https://www.biolegend.com/en-us/products/apc-anti-mouse-human-il-5-antibody-989
26. CD8 (SK1, 344724): https://www.biolegend.com/en-us/products/alexa-fluor-700-anti-human-cd8-antibody-9062
27. CD279 (PD-1) (EH12.2H7, 329952): https://www.biolegend.com/en-us/products/alexa-fluor-700-anti-human-cd279-pd-1-antibody-12365
28. KLRG1 (MAFA) (2F1/KLRG1, 138426): https://www.biolegend.com/en-us/products/apc-cyanine7-anti-mouse-human-klrg1-mafa-antibody-12486
29. IFN-γ (4S.B3, 502530): https://www.biolegend.com/en-us/products/apc-cyanine7-anti-human-ifn-gamma-antibody-6965

Eukaryotic cell lines

Policy information about cell lines and Sex and Gender in Research

Cell line source(s): NIH/3T3, Nalm6, and HEK293T cell lines originally obtained from the American Type Culture Collection (ATCC).

Authentication: Cell lines were authenticated using STR profiling at least once every 3 years from receipt.

Mycoplasma contamination	All cell lines were tested negative for mycoplasma contamination.
Commonly misidentified lines (See ICLAC register)	No commonly misidentified cell lines were used in this study.

Animals and other research organisms

Policy information about studies involving animals; ARRIVE guidelines recommended for reporting animal research, and Sex and Gender in Research

Laboratory animals	Six-week-old NOD/SCID/IL-2Rγnull (NSG) mice were procured from Charles River Laboratory (Lyon, France). All mice were housed in the Center of PhenoGenomics (CPG) animal facility at EPFL, kept in individually ventilated cages at 19-23°C with 45-65% humidity, and maintained on a 12-hour dark/light cycle.
Wild animals	The study did not involve wild animals.
Reporting on sex	Sex was not considered in study design.
Field-collected samples	The study did not involve samples collected in the field.
Ethics oversight	Experimental procedures in mouse studies were approved by the Swiss authorities (Canton of Vaud, animal protocol ID 3533) and performed in accordance with the guidelines from the CPG of EPFL.

Note that full information on the approval of the study protocol must also be provided in the manuscript.

Clinical data

Policy information about clinical studies
All manuscripts should comply with the ICMJE guidelines for publication of clinical research and a completed CONSORT checklist must be included with all submissions.

Clinical trial registration	ClinicalTrials.gov number, NCT01626495, NCT02906371
Study protocol	https://clinicaltrials.gov/ProvidedDocs/95/NCT01626495/Prot_SAP_000.pdf<br>https://clinicaltrials.gov/ct2/show/NCT02906371
Data collection	This study is not a clinical study but uses biospecimens collected under the aforementioned clinical trials. Data collection occurred at the times indicated in the manuscript (from September 2012 to July 2022) at the University of Pennsylvania and Children's Hospital of Philadelphia.
Outcomes	This study is not a clinical study but uses biospecimens collected under the aforementioned clinical trials. Primary and secondary outcomes can be found in the above clinical study and protocol.

Flow Cytometry

Plots

Confirm that:

☒ The axis labels state the marker and fluorochrome used (e.g. CD4-FITC).

☒ The axis scales are clearly visible. Include numbers along axes only for bottom left plot of group (a 'group' is an analysis of identical markers).

☒ All plots are contour plots with outliers or pseudocolor plots.

☒ A numerical value for number of cells or percentage (with statistics) is provided.

Methodology

Sample preparation	For flow cytometry analysis of CD19-3T3 stimulated CAR T cells from 82 ALL patients, the cocultured cells underwent a series of processing steps for immunostaining. Initially, they were washed twice in PBS and then stained for 20 minutes at room temperature (RT) with Live Dead Blue detection reagent (Thermo Fisher Scientific, Cat# L34962), diluted to 1:800 in PBS, to assess cell viability. Following this, cells were washed twice in FACS staining buffer and subsequently stained for surface molecules for 20 minutes at RT. To fix the stained cells, the Cytofix/CytoPerm Fixation/Permeabilization Kit (BD Biosciences, Cat# 554714) was utilized for 20 minutes at RT, while being protected from light. Subsequently, cells were washed twice with 1x Perm/Wash buffer and then stained for CAR19 and intracellular cytokines using antibodies in Perm/Wash buffer. This staining process was carried out for 20 minutes at RT in the dark. Then, cells underwent two additional washes with Perm/Wash buffer before being re-suspended in FACS staining buffer for subsequent analysis. Cell-surface antibodies were used at a 1:100 dilution during staining, and intracellular antibodies at a 1:50 dilution. Samples were run on the Cytek Aurora and analysis was performed using FlowJo v10.8.0.<br><br>For flow cytometry analysis of CAR T cells isolated from mouse peripheral blood, mouse blood (50µL) was collected from the

tail at specified time points for peripheral CAR T cell analysis. The collected samples were resuspended in PBS with EDTA (2mM), and the red blood cells were removed using ACK lysis buffer (Gibco, Cat# A1049201). For surface marker staining, cells were incubated with an antibody panel at 4°C for 30 minutes, followed by live/dead staining using Zombie Aqua Fixable Dye (BioLegend, Cat# 423101). Cells were then washed and resuspended in PBS with 0.2% BSA for flow cytometry analysis. Intracellular cytokine staining was performed by first stimulating cells with a Cell Stimulation Cocktail (Invitrogen, Cat# 00-4970-03) for 5 hours at 37°C to induce cytokine production. Subsequently, cells were stained for surface markers and live/dead dye as previously described, then fixed and permeabilized using a Cytofix/Cytoperm Kit (BD Biosciences). Intracellular staining with the indicated antibody panel was conducted following the manufacturer's protocol. Cell-surface antibodies were used at a 1:100 dilution during staining, intracellular antibodies at a 1:50 dilution, and live/dead staining at a 1:1,000 dilution. Data were collected using an Attune NxT Flow Cytometer with Attune NxT Software v.3 (Invitrogen) and analyzed using FlowJo 10.6.1 (Tree Star).

Instrument

Samples were run on the Cytek Aurora or Attune NxT Flow Cytometer.

Software

Data were analyzed using FlowJo v10.8.0 or v10.6.1.

Cell population abundance

Post-sort purity was evaluated for all the samples and confirmed prior to data analysis.

Gating strategy

For flow cytometry analysis of CD19-3T3 stimulated CAR T cells from 82 ALL patients, the gating strategy is described in Extended Data Fig. 5a, with gates drawn using FMO controls: Live cells (Live Dead Blue-); Non-monocyte Non-B-cell (CD3+CD14-CD19-); Lymphocytes (FSC-A vs. SSC-A); Singlets (FSC-A vs. FSC-H); CAR19+ cells (CAR19 vs. SSC-A); CD4+ or CD8+ CAR (CD4 vs. CD8).

For flow cytometry analysis of CAR T cells isolated from mouse peripheral blood, we used standard gating strategies: Lymphocytes (FSC-A vs. SSC-A); Singlets (FSC-A vs. FSC-H); Live cells (fixable Aqua dye signals); CAR19+ cells (CD3+CAR19+). Gate margins were determined by isotype controls and fluorescence-minus-one controls.

☒ Tick this box to confirm that a figure exemplifying the gating strategy is provided in the Supplementary Information.
